# Colocalized radiofrequency and pulsed field ablation of incessant ventricular tachycardia with suspected intramural circuit in ischaemic cardiomyopathy using a 3.5 mm single-tip catheter under eCPR: a case report

**DOI:** 10.1093/ehjcr/ytag225

**Published:** 2026-03-20

**Authors:** Martin Borlich, Susann Groschke, Maryuri Lisbeth Delgado López, Stephan Fichtlscherer, Leon Iden

**Affiliations:** Heart and Vascular Center, Segeberger Kliniken GmbH, Am Kurpark 1, 23795 Bad Segeberg, Germany; Heart and Vascular Center, Segeberger Kliniken GmbH, Am Kurpark 1, 23795 Bad Segeberg, Germany; Heart and Vascular Center, Segeberger Kliniken GmbH, Am Kurpark 1, 23795 Bad Segeberg, Germany; Heart and Vascular Center, Segeberger Kliniken GmbH, Am Kurpark 1, 23795 Bad Segeberg, Germany; Heart and Vascular Center, Segeberger Kliniken GmbH, Am Kurpark 1, 23795 Bad Segeberg, Germany

**Keywords:** Ventricular tachycardia, Ischaemic cardiomyopathy, Pulsed-field ablation, Radiofrequency catheter ablation, Dual-energy ablation, Energy stacking, Veno-arterial extracorporeal membrane oxygenation (VA-ECMO), Case report

## Abstract

**Background:**

Incessant ventricular tachycardia (VT) in advanced ischaemic cardiomyopathy is a life-threatening condition, particularly when maintained by deep intramyocardial scar channels that can be difficult to eliminate with conventional radiofrequency (RF) ablation. Pulsed-field ablation (PFA) is a non-thermal, myocardium-selective modality with the potential to target arrhythmogenic tissue while minimizing collateral injury.

**Case summary:**

We report a case of incessant VT in a 60-year-old man with severe ischaemic cardiomyopathy in whom acute VT termination and final non-inducibility were achieved using a sequential dual-energy strategy combining RF and PFA delivered with a 3.5 mm open-irrigated catheter capable of both modalities [Dual Energy THERMOCOOL SMARTTOUCH™ (DE-STSF), Johnson & Johnson MedTech]. The procedure was performed under extracorporeal cardiopulmonary resuscitation (eCPR) via veno-arterial extracorporeal membrane oxygenation (VA-ECMO) during electrical storm. Despite VT termination and arrhythmia control, the patient died on Day 5 from refractory shock with progressive multiorgan failure.

**Discussion:**

This case supports the feasibility of colocalized sequential RF–PFA (‘energy stacking’) as an adjunct strategy for suspected intramural post-infarction VT substrate in selected high-risk patients. Supported by VA-ECMO, this dual-energy strategy achieved acute VT non-inducibility and arrhythmia control, despite an unfavourable overall clinical outcome. Further systematic evaluation is warranted to define the incremental role of dual-energy lesion delivery in ventricular substrates.

Learning points

- eCPR with VA-ECMO can create a haemodynamic bridge to emergent catheter ablation in refractory cardiac arrest due to electrical storm.
- In suspected deep septal or intramural post-infarction VT, sequential colocalized RF plus pulsed-field ablation may improve acute VT termination when RF alone is insufficient.

## Introduction

Ventricular tachycardia in the setting of ischaemic cardiomyopathy (ICM) is typically scar-related and may involve deep septal or intramural components that are difficult to eliminate with standard endocardial radiofrequency (RF) ablation. High-resolution human mapping studies suggest that a significant component of the ventricular tachycardia (VT) circuit involves intramural myocardium rather than being purely endocardial or epicardial.^1^ The ablation success is often limited when critical components of the VT circuit involve deep septal or intramural myocardium, beyond the effective lesion depth of standard endocardial radiofrequency (RF) ablation. Pulsed-field ablation (PFA), based on irreversible electroporation, produces non-thermal cell death and is highly selective for cardiomyocytes, with relative sparing of collateral structures such as coronary arteries, valves, nerves, or smooth muscle. Experimental and early clinical data suggest that PFA generates homogeneous lesions with minimal inflammation and may complement RF in hybrid approaches. Histopathological human data have demonstrated comparable lesion depth for single PFA and RF applications, but importantly, the potential of stacked, sequential applications to create deeper or more transmural tissue effect is given.^2^ We here present the case of a 60-year-old man with severe ICM, recurrent electrical storm, and cardiogenic shock who underwent RF/PF sequential ablation (‘dual energy’, ‘energy stacking’) of a left ventricular septal substrate while on veno-arterial extracorporeal membrane oxygenation (VA-ECMO), resulting in successful termination of incessant VT.

## Summary figure

**Figure:**
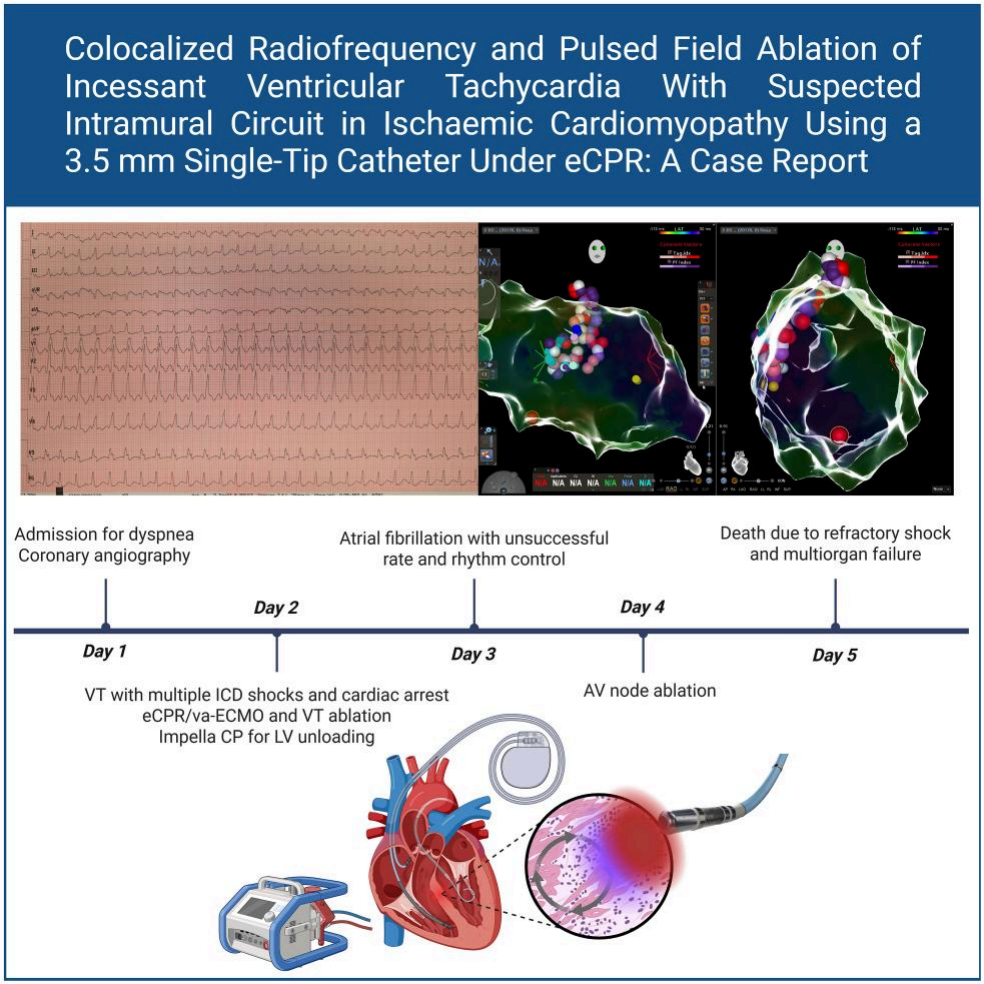


## Case presentation

A 60-year-old man with severe ischaemic cardiomyopathy (left ventricular ejection fraction: 23%) presented with progressive dyspnoea and was admitted for elective coronary angiography. His medical history included an anterior ST-segment elevation myocardial infarction (STEMI) in 2012 due to proximal left anterior descending (LAD) coronary artery occlusion treated with primary percutaneous coronary intervention (PCI) and drug-eluting stent (DES), residual chronic diffuse three-vessel coronary artery disease (CAD), chronic kidney disease Acute Kidney Injury Network (AKIN) stage III, paroxysmal atrial fibrillation, and implantable cardioverter-defibrillator (ICD) implantation in 2013 for primary prevention (generator change in 2021). He had undergone prior catheter ablation for high-burden premature ventricular complexes (PVC) in June 2025 targeting fascicular PVCs with three different exit sites, consistent with a focal/Purkinje-related mechanism. At baseline, the patient was receiving guideline-directed medical therapy for heart failure with reduced ejection fraction (HFrEF) including metoprolol succinate, sacubitril/valsartan, dapagliflozin, eplerenone, vericiguat, and torasemide and was treated with amiodarone as antiarrhythmic therapy. Coronary angiography ruled out relevant *de novo* high-grade stenosis and confirmed a stable coronary status.

**Figure 1:**
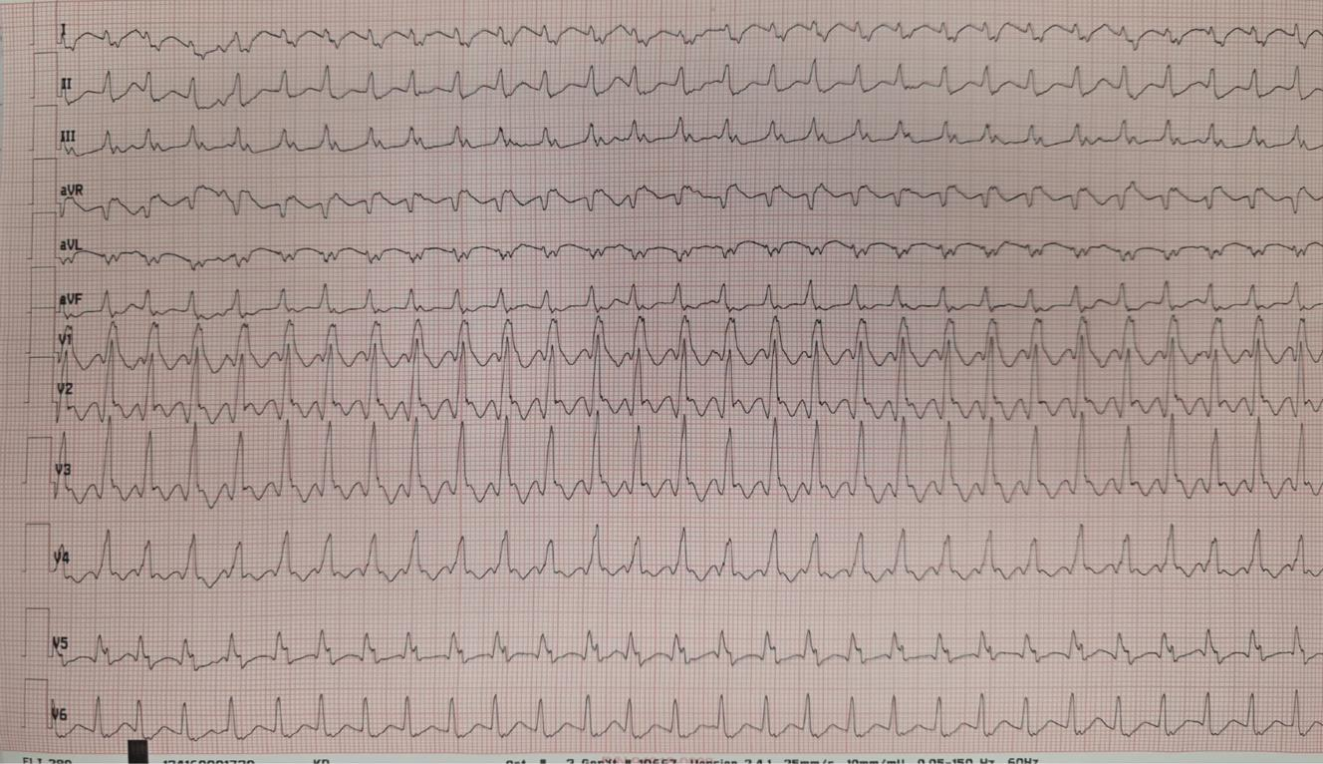
ECG of the ventricular tachycardia (25 mm/s, 10 mm/mV).

**Figure 2:**
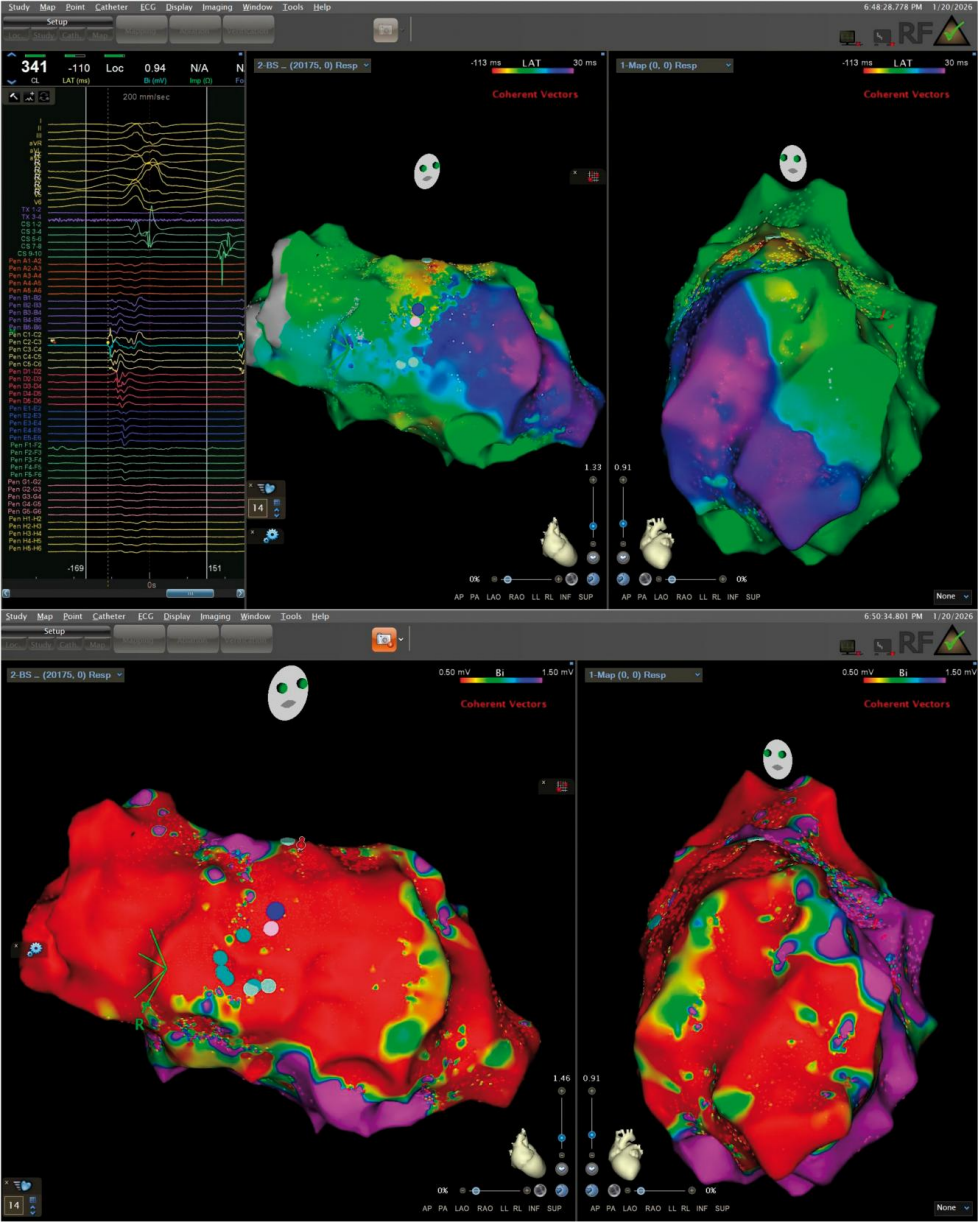
*(A)* Local activation time (LAT) map of ventricular tachycardia in right anterior oblique cranial and left anterior oblique projections. Electrograms from the OCTARAY™ catheter at the site of earliest activation are displayed, consistent with a deep intramural septal substrate. *(B)* Corresponding bipolar voltage map in the same projections, interpreted using standard cut-offs, with dense scar defined as <0.5 mV and border-zone tissue as <1.5 mV.

On the day after coronary angiography, while still in hospital, he developed cardiac arrest due to sustained monomorphic ventricular tachycardia (VT) with multiple ICD shocks and rapid VT re-onset within seconds (*Figure 1*). Cardiopulmonary resuscitation was performed and VA-ECMO was initiated via right femoral access. Because of recurrent monomorphic VT despite lidocaine, magnesium, and amiodarone, emergent catheter ablation was undertaken directly after VA-ECMO cannulation in line with the European Society of Cardiology Guidelines for the management of patients with ventricular arrhythmias and the prevention of sudden cardiac death.^3^ Under sustained VT (QRS = 150 ms) with a CL of 360 ms, RBBB morphology, inferior axis and positive concordance in V1–V6, left femoral venous access was obtained and a decapolar catheter (WEBSTER™ CS catheter, Biosense Webster, Johnson & Johnson MedTech) was positioned in the coronary sinus, documenting VA dissociation and confirming VT. Transseptal puncture was performed, and electroanatomical mapping was performed using the CARTO™ 3 system (Version 8.5; Biosense Webster, Johnson & Johnson MedTech) with an OCTARAY™ mapping catheter (D-curve; 2–2–2 mm spacing). Ultra-high-density mapping of the left ventricle during ongoing VT showed only ∼40% of the tachycardia cycle length (*Figure 2*), with presystolic potentials at the earliest endocardial activation site and additional presystolic electrograms along a septal scar, suggesting slow-conduction channels and raising suspicion of a deeper septal substrate component; therefore, we elected to deliver colocalized sequential RF followed by PFA upfront to maximize the likelihood of acute VT termination.^2^ Entrainment mapping was not performed. Ablation was delivered using the 7.5Fr 3.5 mm saline-irrigated Dual Energy THERMOCOOL SMARTTOUCH™ SF catheter (F-curve; unidirectional) connected to the TRUPULSE 2™ generator (Johnson & Johnson MedTech) enabling delivery of both RF and pulsed-field energy.

**Figure 3:**
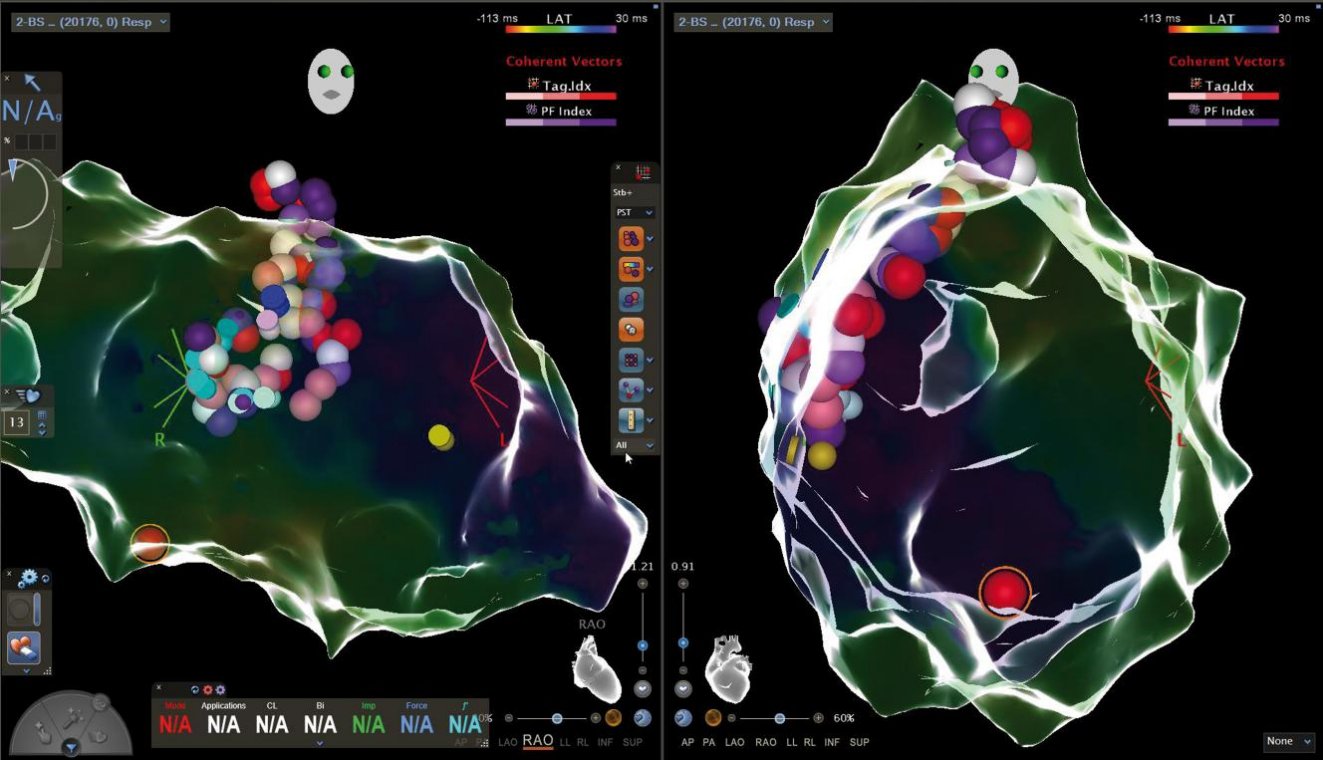
Final lesion set with colocalized RF (red) and PFA (purple) applications across septal substrate (RAO and LAO projection of the LV).

At each target site, RF ablation (50 W, AI-guided; target AI 600) was delivered first, immediately followed—without catheter movement—by a colocalized PFA application (biphasic, 1800 V peak-to-peak, monopolar, microsecond sinusoidal waveforms between the ablation electrode and a skin patch during saline irrigation of the ablation electrode at 4 ml/min, up to 24 burst pulses). This sequential RF–PFA ‘energy stacking’ strategy was applied along the slow-conduction channels and regions of late or fractionated potentials within the septal scar (*Figure 3*). Energy delivery at the earliest activation site followed by further lesions towards the previously identified presystolic potentials resulted in VT termination, accompanied by the development of complete left bundle branch block. After homogenization of the target area with combined RF/PF applications, VT was non-inducible with up to three extrastimuli from both the LV apex and a septal pacing site adjacent to the treated region. The final lesion set in RAO and LAO projections showed tightly clustered RF and PFA applications, particularly across the basal inferior septum.

Following arrhythmia control, predominantly laminar arterial flow was observed and an Impella CP™ device (Abiomed/Johnson & Johnson MedTech) was implanted for left ventricular unloading in addition to VA-ECMO. On the following day, pulsatile flow was restored and vasoactive support was markedly reduced. On Day 3, the patient developed new-onset atrial fibrillation with rapid ventricular response (∼160 b.p.m.) causing haemodynamic deterioration refractory to repeated cardioversion and class III antiarrhythmic boluses. A joint decision for His-bundle ablation was made and performed the same day, resulting in permanent VVI pacing at 75 b.p.m.

Two cranial CT scans showed no macroscopic evidence of cerebral ischaemia. From Day 4 onwards, progressive multiorgan failure with worsening hepatic and renal function and rhabdomyolysis developed, attributed to prolonged cardiogenic shock and high-dose vasopressor therapy. After discussion with the family, further escalation of care was withheld and the patient died from electromechanical dissociation on Day 5.

## Discussion

This case illustrates several important aspects in the management of incessant VT in advanced ischaemic cardiomyopathy. Deep septal or intramyocardial re-entry circuits may drive electrical storm and are difficult to eliminate with conventional endocardial RF ablation alone because of limited lesion depth and safety concerns, particularly near the conduction system and coronary arteries. Mechanical circulatory support with VA-ECMO, and when needed additional left ventricular unloading, can stabilize haemodynamic and create a window to perform detailed mapping and ablation in accordance with contemporary guideline recommendations.^3^ Although the index infarction was treated with early reperfusion, advanced ischaemic cardiomyopathy with septal scar can still harbour deeper conducting channels; in the present case, any intramural involvement remains a hypothesis in the absence of imaging and complete circuit delineation. Due to the extreme clinical situation of ongoing eCPR with refractory VT and necessity of high-dose anticoagulants due to the ongoing ECMO therapy, primary endocardial ablation was performed without epicardial puncture with the perspective of full delineation of the entire VT circuit.

Pulsed-field ablation is a non-thermal, myocardium-selective energy source that appears to spare adjacent non-cardiac structures and to create sharply demarcated, homogeneous lesions with minimal inflammatory reaction. Experimental and early clinical data suggest that when RF and PF lesions are applied sequentially at exactly the same site, their effects may be complementary, potentially extending lesion depth or transmurality while preserving a favourable safety profile.^2,4–6^ Energy stacking using PFA after RF ablation may overcome the compromised ability of RFA to form deep lesions in areas of scar tissue.^7^

Recent early clinical experience suggests the technical feasibility of using dual-energy lattice-tip catheter platforms for ventricular tachycardia mapping and substrate modification employing both radiofrequency and pulsed-field energy. Notably, Mannion and Lyne recently reported a *de novo* VT ablation using stacked RF and pulsed-field applications via a dual-energy lattice-tipped catheter, applying pulsed-field energy over prior RF lesions with acute procedural success.^8^ These emerging data support the concept that sequential dual-energy lesion delivery may enhance tissue effect in ventricular substrate and provide a relevant context for the present case using a non–lattice-tip dual-energy catheter platform.

Alternative strategies to address deep septal or intramural VT substrate include bipolar ablation across the septum,^9^  *trans*-coronary ethanol ablation,^10^ needle catheter ablation,^11^ and adjunctive lesion-deepening approaches such as half-normal saline irrigation.^12^ These techniques may increase lesion depth but are associated with increased procedural complexity and centre-specific availability and should be considered on an individual basis depending on anatomy, substrate location, and clinical urgency.

In this patient, colocalized sequential RF/PF ablation along septal slow-conduction channels rendered VT non-inducible despite previously incomplete activation mapping, supporting the concept of ‘energy stacking’ for suspected intramural post-infarction VT circuits. Although the patient ultimately died from refractory multiorgan failure, the arrhythmia was successfully controlled and no further VT occurred. Death occurred due to refractory shock with progressive multiorgan failure following prolonged cardiogenic shock and high-dose vasopressor therapy and was not attributable to recurrent ventricular arrhythmia. Dual-energy ablation using a single catheter may therefore represent a promising option for carefully selected high-risk patients with intramural VT substrate, warranting further systematic evaluation.

Limitations of this report include incomplete activation mapping during incessant VT (∼40% of the tachycardia cycle length), the absence of entrainment or pace mapping, no epicardial assessment, and no post-ablation remapping or imaging (ICE or CMR) to verify circuit anatomy or lesion depth. Therefore, intramural involvement and any incremental efficacy of colocalized RF–PFA lesion delivery should be interpreted as a hypothesis rather than a demonstrated mechanism.

To the best of our knowledge, this is the first reported case of VT ablation using the Dual Energy THERMOCOOL SMARTTOUCH™ SF catheter (DE-STSF, Johnson & Johnson MedTech, Diamond Bar, CA, USA).

In summary, sequential delivery of RF and PFA using a dual-energy catheter achieved acute termination and non-inducibility of an incessant septal VT in a patient with severe ischaemic cardiomyopathy under full mechanical circulatory support. This ‘energy stacking’ approach may represent a feasible adjunct strategy in selected high-risk patients with suspected intramural VT substrate. Further systematic evaluation is warranted to define its incremental role in ventricular substrates.

## Lead author biography


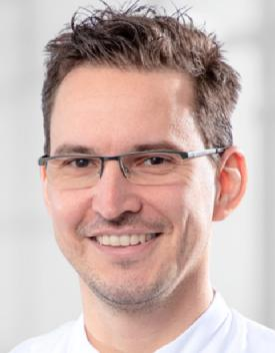


Martin Borlich, MD is a consultant cardiologist and electrophysiologist at the Heart and Vascular Center, Segeberger Kliniken, Germany. He studied medicine at the University of Lübeck and received his MD in 2014. He completed his training in internal medicine and cardiology at Segeberger Kliniken and undertook a fellowship in invasive electrophysiology at Heart Center Leipzig in 2017. His main clinical and research interests include catheter ablation of complex atrial and ventricular arrhythmias and fluoroscopy reduction techniques.

## Data Availability

The data underlying this case report are available from the corresponding author upon reasonable request, subject to ethical and privacy considerations.

## Notes

**Consent:** Written informed consent was obtained permitting use of the relevant clinical and procedural data for publication; all data were anonymized to prevent patient identification.
